# Innovation in daily hemodialysis: high-flux vs. hemodiafiltration

**DOI:** 10.1590/2175-8239-JBN-2026-E003en

**Published:** 2026-03-06

**Authors:** Dirceu Reis da Silva

**Affiliations:** 1 Hospital de Clínicas de Porto Alegre, Porto Alegre, RS, Brazil.

Hemodialysis, as currently practiced, is anchored in principles that, especially regarding session frequency and duration, are largely empirical in origin. Even so, it represents one of the most remarkable scientific and social achievements of the twentieth century in the field of health. The term “dialysis” originally referred to laboratory processes of separation using semipermeable membranes, evolving from the beginning of the twentieth century until, only in the 1960s, it became a clinical procedure widely applicable to humans. This transformation resulted from complementary advances, such as the development of viable vascular access, the use of heparin as an anticoagulant, and the design of the first filters, which allowed extracorporeal blood purification to be consolidated as an effective clinical therapy with major social impact.

The frequency of three weekly sessions, with an average duration of four hours, was established empirically once satisfactory relief of uremic, congestive, and neurological symptoms was achieved, in addition to the control of hyperkalemia. Subsequently, adjustments were introduced based on the technological evolution of dialyzers, increases in blood flow, and, later, the incorporation of the Kt/V concept described in the 1980s^1^. As public and private payers consolidated these standards, the three-times-weekly hemodialysis regimen became the predominant model on a global scale.

Over the years, innovative experiences have sought to refine this model, including the use of filters with different characteristics, the incorporation of convective transport (with greater clearance of middle molecules), the development of home dialysis, and the adoption of regimens with greater variability in duration, frequency, and intensity of blood and dialysate flows. In particular, hemodiafiltration (HDF) has been widely adopted in Europe and Asia, as well as increasingly in Brazil, with the definition of a minimum convective volume requirement of 23 L/1.73 m^2^ to demonstrate its clinical benefits, the use of ultrapure water, and higher blood flow rates^2,3^. With HDF, a significant impact has been obtained on the optimization of phosphorus and middle-molecule clearance, treatment tolerance, quality-of-life outcomes, and mortality^2^, as well as a potential effect on reducing oxidative stress and inflammatory processes^4^. The mitigation of inflammation appears to be associated with lower hepcidin levels^5^, better control of anemia with reduced erythropoietin use, improved quality of life, and a possible reduction in cardiovascular morbidity and mortality.

Given the known limitations of Kt/V as the main indicator of dialysis adequacy^6,7^ and the growing diversity of prescriptions and modalities, the development of more comprehensive tools for the assessment of therapy has become essential. Such measures must be able to compare the effectiveness of different therapeutic models, in line with the multidimensional assessment approach advocated by Perl et al.^7^

In the study by Melo et al.^8^, included in this issue, the authors describe the experience of employing hemodiafiltration in the context of short daily sessions, and there is a clear attempt to go beyond Kt/V, reflecting a more integrated, patient-centered paradigm.

Despite the assessment of outcomes in a limited group of patients from a single center, the primary objective is to demonstrate the safe transition between the two blood purification modalities. Preliminary findings suggest some reduction in myoglobin and HDL cholesterol levels, as well as a benefit in anemia control. Other parameters did not differ between the study periods, pending further exploration of these findings in larger populations and other settings. In any case, the evidence encourages the replication of these evaluations, since, for example, reductions in serum phosphate^6^ are well documented in high-flux hemodialysis regimens with short and frequent sessions, as well as in HDF. Studies using these techniques in combination, with larger sample sizes and longer exposure times, are awaited to confirm clinical benefits. The authors are also noteworthy for their effort to develop and apply appropriate tools for the assessment of clearance in this very specific context.

Patient-centered care—a structuring principle of modern clinical management^9,10^—reinforces the importance of multiple dialysis regimen options to reconcile clinical needs with individual values, beliefs, and lifestyles. In this context, engaging patients in a personalized dialysis model is not merely a technical refinement but rather an essential strategy to promote greater treatment adherence and patient satisfaction.

Considering the single-center design, the small number of participants, and the unique conditions of application (short and frequent treatments, the use of ultrapure water, and the use of high-flux, biocompatible filters), it is understandable that robust differences between modalities are difficult to demonstrate. Nonetheless, there is a broad rationale that points to promising opportunities, and, for this reason, the described experience offers valuable insights and stimulates new reflections on the future of dialysis therapy.

## Data Availability

No new data were generated or analyzed in this study.
