# Erratum: Innovation in daily hemodialysis: high-flux vs. hemodiafiltration

**DOI:** 10.1590/2175-8239-JBN-2026-E003ener

**Published:** 2026-03-17

In the article “Innovation in daily hemodialysis: high-flux vs. hemodiafiltration”, with DOI code number: https://doi.org/10.1590/2175-8239-JBN-2026-E003en, published in the journal Brazilian Journal of Nephrology, 48(1):e2026E003, 2026, in the page 2:

Where it was written:

8. Melo NCV, Isoni LMRM, Simões RED, Allemand LDS, Vasconcelos DF, Lauar JP, et al. Potential benefits of converting from short daily high-flux hemodialysis to short daily online hemodiafiltration: a pilot study. Braz J Nephrol. 2025;48(1):e20250127.

Should read:

8. Melo NCV, Isoni LMRM, Simões RED, Allemand LDS, Vasconcelos DF, Lauar JP, et al. Potential benefits of converting from short daily high-flux hemodialysis to short daily online hemodiafiltration: a pilot study. Braz J Nephrol. 2026;48(1):e20250127.

Prof. Dr. Miguel Carlos Riella

Editor-in-chief

