# Supplementary material for: Innovation in daily hemodialysis: high-flux vs. hemodiafiltration
Source: J Bras Nefrol. 2026 Mar 6;48(1):e2026E003. doi: 10.1590/2175-8239-JBN-2026-E003en (PMC12965626; doi:10.1590/2175-8239-JBN-2026-E003en)
Supplement: Translation

# Translation: Inovação na hemodiálise diária: alto fluxo vs. hemodiafiltração.

**DOI:** 10.1590/2175-8239-JBN-2026-E003pt

**Authors:** Dirceu Reis da Silva

**Affiliations:** 1 Hospital de Clínicas de Porto Alegre, Porto Alegre, RS, Brasil.

A hemodiálise, tal como atualmente praticada, ancora-se em fundamentos que, especialmente no que diz respeito à frequência e à duração das sessões, têm origem empírica. Ainda assim, constitui uma das mais notáveis conquistas científicas e sociais do século XX no campo da saúde. O termo “diálise” designava originalmente processos laboratoriais de separação por meio de membranas semipermeáveis, evoluindo desde o início do século XX até tornar-se, somente na década de 1960, um procedimento clínico largamente aplicável em seres humanos. Essa transformação resultou de avanços complementares, como o desenvolvimento de acessos vasculares viáveis, a utilização de heparina como anticoagulante e a concepção dos primeiros filtros, o que permitiu consolidar a hemodepuração extracorpórea como terapia clínica efetiva e de grande impacto social.

A frequência de três sessões semanais, com duração média de quatro horas, foi sendo estabelecida empiricamente, à medida que se alcançava alívio satisfatório de sintomas urêmicos, congestivos e neurológicos, além do controle da hiperpotassemia. Posteriormente, ajustes foram introduzidos com base na evolução tecnológica dos dialisadores, no aumento do fluxo sanguíneo e, posteriormente, na incorporação do conceito de Kt/V descrito na década de 1980^1^. À medida que agentes financiadores — públicos e privados — consolidavam esses padrões, o regime de hemodiálise com três sessões semanais tornou-se o modelo predominante em escala global.

Ao longo dos anos, experiências inovadoras buscaram aperfeiçoar esse modelo, incluindo o uso de filtros de diferentes características, a incorporação de transporte convectivo (com maior depuração de moléculas médias), o desenvolvimento da diálise domiciliar e a adoção de regimes com maior variabilidade de duração, frequência e intensidade dos fluxos sanguíneo e de dialisato. Em especial, a hemodiafiltração (HDF) tem sido adotada amplamente na Europa e na Ásia, bem como, de forma crescente, no Brasil, com a delimitação da necessidade de volume convectivo mínimo de 23 L/1,73 m^2^ para a demonstração de seus benefícios clínicos, além do emprego de água ultrapura e da utilização de fluxos de sangue mais elevados^2,3^. Com a HDF, tem-se obtido impacto significativo na otimização da depuração de fósforo e de moléculas médias, na tolerância ao tratamento e nos desfechos de qualidade de vida e de mortalidade^2^, além do potencial efeito na redução do estresse oxidativo e de processos inflamatórios^4^. A mitigação da inflamação parece estar associada a menores níveis de hepcidina^5^, a um melhor controle da anemia, com menor uso de eritropoietina, à otimização da qualidade de vida e à possível redução da morbimortalidade cardiovascular.

Diante das limitações conhecidas do Kt/V como principal indicador de adequação dialítica^6,7^, e da crescente diversidade de prescrições e modalidades, torna-se essencial o desenvolvimento de instrumentos mais abrangentes para a avaliação da terapia. Tais medidas devem ser capazes de comparar a efetividade de modelos terapêuticos distintos, alinhando-se à proposta de avaliação multidimensional defendida por Perl et al.^7^ No estudo de Melo et al.^8^, presente neste volume, descrevese a experiência de empregar hemodiafiltração no contexto de sessões cotidianas de curta duração, observando-se a tentativa de ir além do Kt/V, o que reflete um paradigma mais integrado e centrado no paciente.

Em que pese a avaliação de resultados de um grupo limitado de pacientes provenientes de um centro único, procura-se demonstrar, antes de mais nada, a transição segura entre as duas modalidades de hemodepuração. Achados preliminares apontam para alguma redução da mioglobina e do nível de colesterol-HDL, bem como o benefício no controle da anemia. Outros parâmetros não se mostraram diferentes entre os períodos estudados, no aguardo da exploração desses achados em maiores populações e em outros contextos. De todo modo, as evidências encorajam a replicar estas avaliações, posto que, por exemplo, reduções da fosfatemia^6^ são notórias em regimes de hemodiálise de alto fluxo, com sessões curtas e frequentes, assim como em HDF, aguardando-se estudos com o emprego destas técnicas em combinação, com o aumento da amostra populacional e com maior tempo de exposição, para a confirmação de benefícios clínicos. Notabilizam-se os autores, ainda, pelo esforço de desenvolver e aplicar ferramentas apropriadas de avaliação da depuração, nesse contexto tão particular.

O cuidado centrado no paciente — princípio estruturante da gestão clínica moderna^9,10^ — reforça a importância de múltiplas opções de regime dialítico, de modo a compatibilizar necessidades clínicas com valores, crenças e estilos de vida individuais. Nesse contexto, o engajamento de pacientes em um modelo personalizado de diálise deixa de ser apenas um refinamento técnico, configurando-se como uma estratégia essencial para promover maior adesão terapêutica e satisfação do paciente.

Considerando as características do desenho unicêntrico, o número reduzido de participantes e as condições singulares de aplicação (tratamento curto e frequente, uso de água ultrapura e de filtros de alto fluxo e biocompatibilidade), entende-se a difícil demonstração de diferenças robustas entre as modalidades. Tem-se, de todo modo, um racional abrangente que sinaliza promissoras oportunidades e, por isso mesmo, a experiência descrita oferece valiosas perspectivas e instiga novas reflexões sobre o futuro da terapia dialítica.

## Data Availability

Nenhum dado novo foi gerado ou analisado neste estudo.
