# Supplementary material for: Erratum: Innovation in daily hemodialysis: high-flux vs. hemodiafiltration
Source: J Bras Nefrol. 2026 Mar 17;48(2):e2026E003er. doi: 10.1590/2175-8239-JBN-2026-E003ener (PMC13126810; doi:10.1590/2175-8239-JBN-2026-E003ener)
Supplement: Translation

# Translation: Errata: Inovação na hemodiálise diária: alto fluxo vs. hemodiafiltração

**DOI:** 10.1590/2175-8239-JBN-2026-E003pter

No artigo “Inovação na hemodiálise diária: alto fluxo vs. hemodiafiltração”, com número de DOI: https://doi.org/10.1590/2175-8239-JBN-2026-E003pt, publicado no periódico Brazilian Journal of Nephrology, 48(1):e2026E003, 2026, na página 2:

Onde se lia:

8. Melo NCV, Isoni LMRM, Simões RED, Allemand LDS, Vasconcelos DF, Lauar JP, et al. Potential benefits of converting from short daily high-flux hemodialysis to short daily online hemodiafiltration: a pilot study. Braz J Nephrol. 2025;48(1):e20250127.

Leia-se:

8. Melo NCV, Isoni LMRM, Simões RED, Allemand LDS, Vasconcelos DF, Lauar JP, et al. Potential benefits of converting from short daily high-flux hemodialysis to short daily online hemodiafiltration: a pilot study. Braz J Nephrol. 2026;48(1):e20250127.

Prof. Dr. Miguel Carlos Riella

Editor-chefe
